# Device-Measured Sedentary Patterns and Physical Activity Before and During the COVID-19 Pandemic

**DOI:** 10.1093/geroni/igab046.1309

**Published:** 2021-12-17

**Authors:** Dori Rosenberg, Mikael Anne Greenwood-Hickman, Jing Zhou, Julie Cooper, David Arterburn, Andrea Cook

**Affiliations:** 1 Kaiser Permanente Washington Health Research Institute, Seattle, Washington, United States; 2 Kaiser Permanente Washington, Seattle, Washington, United States; 3 kaiser Permanente Washington Health Research Institute, Seattle, Washington, United States

## Abstract

Little is known about objective levels of sitting time (ST), patterns of ST, and physical activity (PA) among older adults before compared to during the COVID -19 pandemic. We used data from the Healthy Aging Resources to Thrive Trial to examine differences in activPAL-assessed ST, standing time, breaks from sitting, and steps in study enrollees prior to March 2020 (N = 97, % female = 60.8, % white = 81.4; Mean BMI = 35.2) compared to post-March 2020 (N = 47, % female = 70.2, % white = 72.3; Mean BMI = 36.1). During the pandemic, participants had higher sitting time (Mean = 11.5 vs. 10.7 hours/day), fewer breaks from sitting (Mean = 40 vs. 44 breaks/day), and fewer steps (Mean = 4441 vs. 5931 steps/day) than prior to the pandemic. Interventions may be needed to support older adults with obesity in recovering losses in time spent physically active.

